# Establishing a Sequencing Method for the Whole Mitochondrial DNA of Domestic Dogs

**DOI:** 10.3390/ani13142332

**Published:** 2023-07-17

**Authors:** Takehito Sugasawa, Yuki Matsumoto, Hui Fang, Tohru Takemasa, Ritsuko Komine, Shinsuke Tamai, Wenchao Gu, Kei Tanaka, Yasuharu Kanki, Yoichiro Takahashi

**Affiliations:** 1 Laboratory of Clinical Examination and Sports Medicine, Department of Clinical Medicine, Faculty of Medicine, University of Tsukuba, 1-1-1 Tennodai, Tsukuba 305-8577, Japan; 2 Department of Sports Medicine Analysis, Open Facility Network Office, Organization for Open Facility Initiatives, University of Tsukuba, 1-1-1 Tennodai, Tsukuba 305-8577, Japan; 3 Research and Developmental Division, Anicom Insurance Inc., 2-6-3 5F Chojamachi, Yokohamashi-Nakaku, Yokohama 231-0033, Japan; 4 Doctoral Program in Sports Medicine, Graduate School of Comprehensive Human Sciences, University of Tsukuba, 1-1-1 Tennodai, Tsukuba 305-8577, Japan; 5 Institute of Health and Sport Sciences, University of Tsukuba, 1-1-1 Tennodai, Tsukuba 305-8577, Japan; 6 Department of Sport Science and Research, Japan Institute of Sports Sciences, 3-15-1 Nishigaoka, Kita-ku, Tokyo 115-0056, Japan; 7 Department of Diagnostic and Interventional Radiology, University of Tsukuba, 1-1-1 Tennodai, Tsukuba 305-8577, Japan; 8 College of Medicine, School of Medicine and Health Sciences, University of Tsukuba, 1-1-1 Tennodai, Tsukuba 305-8577, Japan; 9 Department of Legal Medicine, Institute of Medicine, University of Tsukuba, 1-1-1 Tennodai, Tsukuba 305-8575, Japan

**Keywords:** *Canis lupus familiaris*, domestic dog, mitochondrial DNA, next-generation sequencing

## Abstract

**Simple Summary:**

The present study aims to establish a method of whole mitochondrial DNA (mtDNA) sequencing for domestic dogs. Prior to proceeding with the experiment, the collection of relevant DNA samples was essential. Therefore, we decided to use oral mucosa DNA, which can be collected non-invasively. A polymerase chain reaction (PCR) was performed using the DNA collected from six dogs raised in Japan and four primer pairs as specific to the mtDNA, after which the amplified products were sequenced using next-generation sequencing. As a result, the whole mtDNA obtained from all dogs was correctly sequenced. Thus, we determined that the method we used in our study may be useful for future research on dog-related medical care and welfare.

**Abstract:**

In human beings, whole mitochondrial DNA (mtDNA) sequencing has been widely used in many research fields, including medicine, forensics, and genetics. With respect to the domestic dog (*Canis lupus familiaris*), which is commonly recognized as being an additional member of the traditional human family structure, research studies on mtDNA should be developed to expand and improve our collective knowledge of dog medicine and welfare as it seems that there is still room for further development in these areas. Moreover, a simple and robust method for sequencing whole mtDNA that can be applied to various dog breeds has not yet been described in the literature. In the present study, we aim to establish such a method for the whole mtDNA sequencing of the domestic dog. In the experiments we conducted, oral mucosa DNA samples obtained from six Japanese domestic dogs were used as a template. We designed four primer pairs that could amplify approximately 5 kbp from each region of the mtDNA and validated several PCR conditions. Subsequently, the PCR amplicons were pooled and subjected to library preparation. The sequencing of the libraries was performed using next-generation sequencing (NGS), followed by bioinformatics analysis. Our results demonstrate that the proposed method can be used to perform highly accurate resequencing. We believe that this method may be useful for future research conducted to better understand dog medicine and welfare.

## 1. Introduction

Mitochondria are organelles that generate energy and play a crucial role in numerous cellular functions, such as ATP production through oxidative phosphorylation (OXPHOS) on respiratory chain (RC) complexes 1 to 5, cellular homeostasis, and apoptosis [1]. Mitochondrial DNA (mtDNA) is the small, circular chromosome found inside mitochondria in mammals, which are cellular organelles located in the cytoplasm [2]. It is typically present at thousands of copies per cell [1,3] and is inherited solely from the mother [2,3]. mtDNA contains 37 genes, with 13 of them providing instructions for producing proteins in the mitochondrial RC complex, which is the site of cellular energy production through the process of OXPHOS [4,5]. Two of the remaining genes code for rRNA and twenty-two for tRNA [4,5]. The standard length of mitochondrial DNA in human beings (*Homo sapiens*) and dogs (*Canis lupus familiaris*; the domestic dog) are 16,569 bp [6] and 16,727 bp [7], respectively, and have been registered in public databases (National Library of Medicine, Bethesda, MD, USA). However, the lengths of these mitochondrial DNA may slightly vary depending on the individual or species. Investigating the mtDNA present in mammals, which serves as the basis of their life support system, has been significantly useful for both medical and genetic research concerning both humans and other animals.

Research on mtDNA was initiated in the late 1970s to investigate the migration history of human beings, culminating in the full sequencing of human mtDNA in the year 1981 [8,9]. The Sanger sequencing method was commonly used in the past to perform mtDNA sequencing, as can be observed in a report previously published in the literature [10]. However, modern research has facilitated the successful establishment of highly accurate and high-throughput mtDNA analysis methods with the advent of next-generation sequencing (NGS) and multiple technological innovations, and this technology is still evolving. There are several research reports available in the literature that have analyzed whole human mtDNA using NGS [11,12,13,14,15,16], with methods ranging from forensic testing, mitochondrial disease testing, cancer diagnostic applications, and psychiatric diagnostic applications [11,12,13,14,15,16]. In addition to medical applications, NGS can also be utilized to explore the migration routes of specific maternal haplogroups and maternal lineages [17]. Given this potential, innovative NGS technologies should also be applicable to the fields of veterinary medicine and animal genetics.

In recent years, domestic dogs have begun to live lifestyles that are similar to those of human beings. In Japan, a survey conducted in 2022 reported that 7.053 million dogs had been bred in the country, which amounted to an average of 1.25 dogs per household [18]. This is a remarkable figure, and it is common to see multiple dogs in public areas. Additionally, the cost of raising and providing medical care for dogs has been increasing annually [18], despite the fact that household incomes have been stagnating. This outcome suggests that domestic dogs are commonly perceived as members of the family, akin to a human child, presenting strong emotional bonds with their owners.

As dogs become more integral to society as pets, dog medicine and welfare will become increasingly important to the public. Therefore, it is essential that the research currently being conducted on dog medicine and genetics is developed in a manner similar to that for human beings. However, those with clinical experience in veterinary medicine have suggested that the research being conducted on dog medicine is not as advanced as that concerning human medicine, particularly in the area of mitochondrial DNA (mtDNA). There is a considerable lack of reports addressing dog mtDNA in comparison to those addressing human mtDNA, indicating the necessity for a shift in the focus of the research. Additionally, two recently published review papers [19,20] addressing the mitochondrial diseases present in dogs demonstrated several cases that could be useful for performing differential diagnoses in veterinary medicine, emphasizing the need to perform an accurate genetic test to elucidate their genetic pathology. Thus, it is necessary to establish a basic method for sequencing the whole length of dog mtDNA in the research.

Ultimately, the aim of the present study was to establish a simple and accessible method for sequencing the whole mtDNA of dogs using NGS and amplicon sequencing methods. Our results present highly accurate resequencing, and we believe that the method proposed in this study has the potential to provide new insights into this research area and facilitate relevant advancements in the research being conducted on dog medicine and welfare.

## 2. Materials and Methods

### 2.1. Research Ethics

This study was approved by the Animal Care Committee, Anicom Specialty Medical Institute Inc. (approval number: 2020-02, 28 September 2020; 2022-02, 29 March 2022). All owners who participated in the study were informed about the protocol of the study, and their consent was obtained. The owners were then asked to collect specimens in the form of oral mucosa. In addition, the owners were instructed in advance by a member of the research team on the most effective method to collect the specimens for this study.

### 2.2. Dogs and Collected Specimens

**Figure 1:**
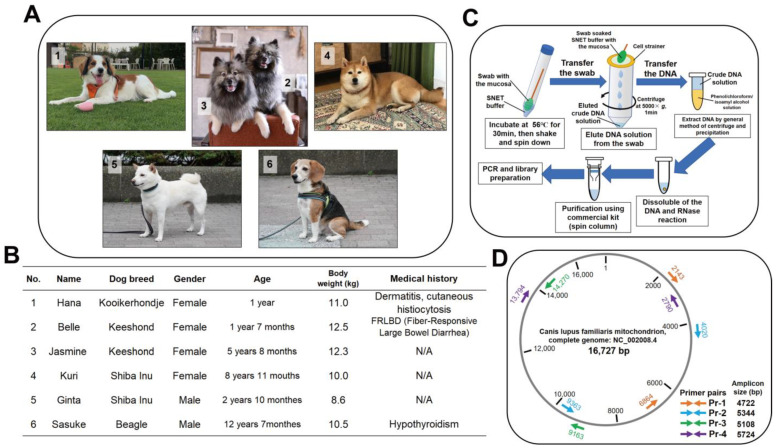
Dogs assigned to this study and the overview of the experiment. (**A**) Pictures of the six dogs from four different breeds; (**B**) basic information about the dogs; (**C**) methods for the extraction and purification of oral mucosal DNA; (**D**) design overview for the primer pairs. Pr: primer pairs; N/A: not applicable.

Six Japanese domestic dogs of four dog breeds were used for this study. The breeds were Kooikerhondje, Keeshond (two dogs; mother and daughter), Shiba Inu (two dogs), and Beagle. The basic information for and photos of these dogs are presented in Figure 1A,B. All dogs were healthy on the day the specimen collection was performed. The oral mucosa was collected by each dog owner by rubbing the dog’s mouth using 10 mm diameter swabs that were 15 cm long (Cat#15162, Hakujuji, Toyoshimaku, Tokyo, Japan). The swabs were immediately immersed in 1 mL of SNET buffer (10 mM Tris-HCl pH 8.0, 100 mM EDTA pH 8.0, 1% SDS, 100 µg/mL Proteinase K) in a 15 mL centrifuge tube and then stored at −20 °C until the day we performed further analyses.

### 2.3. Extraction of Oral Mucosa DNA

An overview of this method is presented in Figure 1C. The swab samples in the SNET buffer, including mucosae, were incubated at 56 °C for 30 min to disassemble the protein, followed by vigorous shaking. Subsequently, the SNET buffer, including mucosae DNA, was eluted from the swab samples by centrifugation at 5000× *g* for 1 min at room temperature, via the use of a cell strainer. The DNA pellets were obtained from 800 µL of the eluted solutions by applying equal volumes of phenol/chloroform/isoamyl alcohol (Cat# 25970-56; Nacalai Tesque, Nakagyo, Kyoto, Japan) with a general centrifugation and precipitation method. The pellets were then dissolved in 50 µL of Milli-Q water containing Ribonuclease A (10 µg/mL) and incubated at 37 °C for 10 min to degrade residual RNA. The solutions were then purified using NucleoSpin Gel and a PCR clean-up kit (Cat# U0609B; Takara Bio, Kusatsu, Shiga, Japan), the latter of which was used according to the manufacturer’s instructions; the final elution volume was 30 µL. The purified DNA solutions were subjected to gel electrophoresis to confirm the degree of degradation with a positive control of intact DNA that was extracted from adipose-derived mesenchymal stem cells, called the 1117 cell (our original), of a Toy Poodle.

### 2.4. Design of Primer Pairs for mtDNA

Four primer pairs (Pr-1 to 4) containing four overlap regions (Figure 1D), which could cover the entire length of the mtDNA, were designed in the experiment using Primer-BLAST [21], a free Web tool. The sequences and other information of the primer pairs are presented in Supplementary Table S1. The predicted amplicon sizes ranged from 4.7 to 5.7 kbp as long-range polymerase chain reactions (LR-PCRs).

### 2.5. Conditions of the LR-PCR

A long-range polymerase chain reaction (PCR) assay was performed to amplify the target regions of each four primer pairs using a KOD One PCR Master Mix reagent (Cat# KMM-101; TOYOBO, Osaka, Japan) and including a high-fidelity PCR enzyme. The template and reagent volume and primer concentrations were 2 and 10 µL and 300 nM, respectively, for a total reaction volume of 20 µL per reaction tube. A negative control sample was also established using Milli-Q water instead of the template. Additionally, a positive control sample was established using the intact DNA of the 1117 cell. The conditions for thermal cycling were as follows: 98 °C for 1 min, 5 cycles of 98 °C for 10 s and 74 °C for 30 s, 5 cycles of 98 °C for 10 s and 70 °C for 30 s, 5 cycles of 98 °C for 10 s and 72 °C for 30 s, 30 cycles of 98 °C for 10 s and 68 °C for 30 s, and 4 °C for ∞. The condition of the thermal cycling was a step-down method that could reduce non-specific amplifications. The four primer PCR reactions were each performed in separate tubes, i.e., four separate amplicons were created from one DNA sample. The PCR amplicons were analyzed on an Agilent 2100 Bioanalyzer (Agilent Technologies, Santa Clara, CA, USA) using a DNA 7500 kit (Cat#5067-1506; Agilent Technologies). Optimal DNA concentrations were also investigated prior to the library preparation of NGS using Sasuke’s DNA (No. 6 in Figure 1A,B).

### 2.6. Library Preparation of NGS

To obtain uniform coverage, each PCR amplicon, including a positive control, was pooled into a single tube with factors calculated from the molar ratio and concentration of the amplicons. Specifically, the following formula was used: factor-1 = predicted amplicon size (bp)/5722; factor-2 = 10/obtained amplicon (nM); each amplicon volume required for the pooling (μL) = 15 × factor-1 × factor-2. Then, a 0.8× suspension of magnetic beads (NucleoMag NGS clean-up and Size Select; Takara Bio) was dispensed into the pooling amplicons and purified according to the manufacturer’s instructions with 30 µL of the final elution volume. Following the purification step, the concentrations of the amplicon DNA samples were measured and then adjusted to 10 ng/μL. The amplicon DNA was used to prepare libraries using a Lotus DNA Library Prep Kit for NGS (Cat# 10001073; Integrated DNA Technologies, Coralville, IA, USA) according to the manufacturer’s instructions. The fragmentation reaction time was 10 min, and there were PCR cycles during the preparation step. The final elution volume of the libraries was 10 μL. Each library concentration was quantified using a qPCR assay with a standard curve, targeting the adapter sequences. TB Green Premix Ex Taq II (Cat# RR820; Takara Bio) with the primer pairs was used on QuantStudio 5 Real-Time PCR Systems (Thermo Fisher Scientific, Waltham, MA, USA) as triplicate measurements. The sequences of the primer pairs were as follows: F-TGATACGGCGACCACCGAGA, R-AAGCAGAAGACGGCATACGA. The template volume and primer concentrations were 2 µL and 100 nM, respectively, for a total reaction volume of 10 µL per well. The conditions for thermal cycling were 95 °C for 5 min, followed by 40 cycles of 95 °C for 2 s and 60 °C for 30 s with a melt-curve stage. Finally, the concentrations of each library were calculated from the standard curve (R^2^ > 0.99). Then, the libraries were adjusted to 10 nM and pooled in an equal volume into one tube.

### 2.7. NGS Rum

The pooled libraries were mixed with the PhiX Control v3 Library (final 10%; Cat# FC-110-3001, Illumina, San Diego, CA, USA), diluted to 1 nM, and then subjected to denaturation and neutralization processes. Subsequently, the libraries were diluted further to 1.4 pM and then applied for an NGS run using a MiniSeq Mid Output Kit (300-cycles) (Cat#FC-420-1004; Illumina) in the MiniSeq System (Illumina). The sequencing was performed with the paired-end reads of 150 bases. The cluster density was 312 K/mm^2^. In addition, the passing filter rate of over Q30 for the clusters was 88.39%, and the data yield was 5.57 G bases with 32.2 M paired-end reads. Overall, the NGS run was considered capable of obtaining high-quality data. Subsequently, FASTQ files were exported following the performance of further bioinformatics analyses.

### 2.8. Bioinformatics Analysis

The basic information of the NGS run data was checked with CLC Genomics Workbench 23.0.1 software (QIAGEN, Hilden, Germany). In the quality assessment of the reads, a PHRED score over 20 was confirmed for 99.3% of all reads, indicating the success of the run. The read numbers were 3.1 to 5.6 M per sample as paired-end reads. The resequencing analysis for the mtDNA was performed following procedures performed on CLC software. First, the obtained FASTQ files were trimmed using the “Trim Reads” command. This operation could eliminate poor-quality reads. Following the trimming step, 99.7% reads remained for each sample. Then, mapping was performed using the “Map Reads to Reference” command with general whole mitochondrial DNA sequences [22], and the mapping rates as paired-end reads were 98.62–99.34%. Subsequently, FASTA files were obtained using the “Extract Consensus Sequence” command. The variants were detected using the “Basic Variant Detection” command following the confirmation of amino acid changes (non-synonymous) by the “Amino Acid Changes” command with a GFF (general feature format) file [22]. The maternal inheritance factor was examined between the obtained mtDNA sequences for dogs No. 2 and 3 (the daughter and mother) using the “Alignment and Trees” command. In addition, BAM files were exported from CLC software. Then, corrasion analysis and principal component analysis (PCA) for the BAM files were performed using the “bamCoberrage”, “plotCorrelation”, and “plotPCA” algorithms on deepTools [23] after creating the index file using SAMtools [24].

The obtained FASTA file and the files of other dog breeds downloaded from the NCBI database [25] were subjected to phylogenetic analysis. We used the MUSCLE alignment program [26] implemented in MEGA X software (version 10.2.4) [27]. Model selection was performed based on the Bayesian information criterion (BIC) scores we obtained, and the lowest BIC scores were considered to be the most appropriate to describe the substitution pattern we observed. A phylogenetic tree was constructed using the neighbor-joining method [28]. To evaluate the reliability of the branches in the tree, bootstrap methods were used with 1000 replications. The Himalayan wolf (*Canis lupus chanco*) was used as an outgroup.

## 3. Results

### 3.1. Oral Mucosal DNA Was Successfully Extracted from and Purified in All Samples

**Figure 2:**
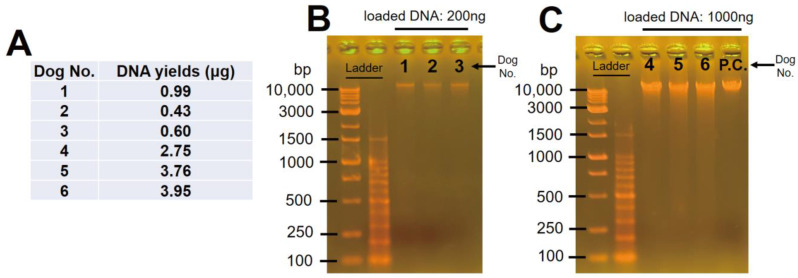
Yields and electropherogram of all samples. (**A**) Yields of the DNA; (**B**) electropherogram of high- and (**C**) low-yield samples. P.C.: positive control.

The DNA samples were successfully extracted from and purified in all of the samples. However, there were samples presenting low (dog No. 1–3; 0.60–0.99 μg) and high (dog No. 4–6; 2.75–3.95 μg) yields (Figure 2A). Depending on the yield we obtained, two separate electrophoresis experiments were performed to verify the degradation value. As a result, the presence of slight smears was confirmed; however, intact bands over 10 kb were also confirmed in all of the positive control samples (Figure 2B,C). Taken together, the DNA samples presented slight degradation while demonstrating that this experimental method was appropriate. Additionally, it was suggested that the DNA samples could be used to amplify whole mtDNA.

### 3.2. Optimal LR-PCR Condition Was Determined, and the High Specificity of Each Primer Pair Was Confirmed

**Figure 3:**
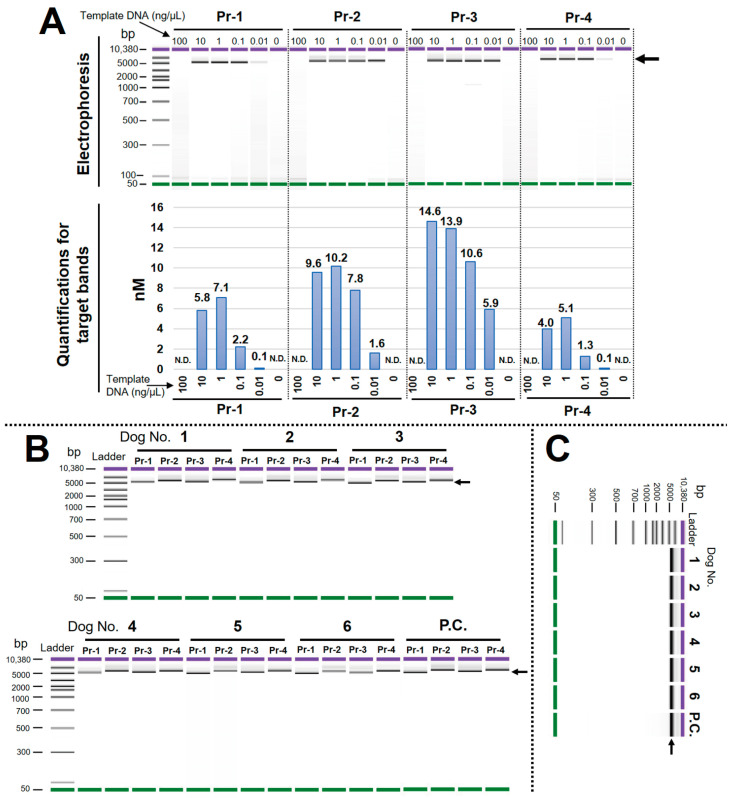
Determination of the optimal concentrations of template DNA and confirmation of the amplicons. (**A**) Validation of template concentrations; (**B**) confirmation of amplicons using all samples and primer pairs; (**C**) confirmation of pooled and purified amplicons. P.C.: positive control. N.D.: not detected. Arrows represent target bands.

The verification of the optimal template concentration was performed in the experiment. As a result, 1 ng/μL (2 ng DNA/20 μL reaction) presented good amplification efficiency without a non-specific band for all primer pairs. On the other hand, amplicons for all primer pairs could not be obtained at 100 ng/μL (200 ng DNA/20 μL reaction) (Figure 3A). Therefore, we decided to use a template concentration of 1 ng/μL for the subsequent experiments. The LR-PCR for all samples and primer pairs presented good amplicons without non-specific amplifications and primer dimers (Figure 3B). In addition, electropherograms performed following amplicon pooling and purification steps also showed a uniform-band pattern in the samples (Figure 3C). These results indicate that the LR-PCR conditions and primer specificity were conducive to achieving the desired outcomes.

### 3.3. Whole Mitochondrial DNA Was Accurately Resequenced in All Dogs

**Figure 4:**
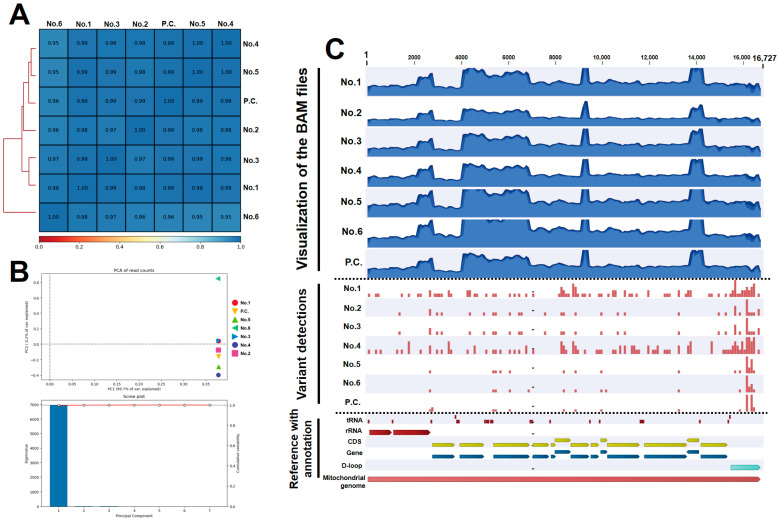
Visualization of the bioinformatics analysis. (**A**) Heat map of Pearson’s correlation analysis on read counts of every 100 bases between the samples; (**B**) PCA; (**C**) visualization of the BAM files and variant locations, based on reference [22], for the whole mtDNA sequences.

Using CLC software, the reads were mapped to the reference of the mtDNA and resequencing was performed. As a result, the whole mtDNA sequences obtained from all samples presented a high quality score. The software used assigned a quality score of 0 to 64 for each base when extracting the base sequences from the mapped read data of the BAM file. The results show a score of 64 for all bases in all of the samples (Supplementary Figure S1), indicating that resequencing was performed with high precision and accuracy. Then, homogeneity coverage was verified on the mapped reads. Every 100-base reads count was highly correlated between the samples (Figure 4A). Additionally, PCA also demonstrated a high similarity among the samples with a PC1 of 99.7% (Figure 4B). These results indicate that an even coverage was obtained on the whole mtDNA sequences without producing a sample-dependent bias. Figure 4C presents a visualization of the BAM files and the variant locations for each sample with the appropriate references. Leads were generally mapped uniformly, except for the four overlap regions of the amplicon; however, a decrease in the coverage was observed in the D-loop region for all samples (Figure 4C, upper illustration). By checking the positions of the variant indicated by the bar plot (red bar), we observed numerous variants present in the D-loop region. Moreover, dogs No. 1 and 4 were confirmed to have more variants than the other dogs throughout the entire region (Figure 4C, middle illustration). A summary of the variants is presented in Table 1 and Supplementary Table S2. Some variants that induced an alteration in amino acids as non-synonymous were observed in each dog; however, none of the changes observed were pathological or replaced as stop codons. Wherever maternal inheritance could be detected, the mtDNA sequences we obtained for dogs No. 2 and 3 (the daughter and mother) were examined by alignment, and we then found that 100% of the sequences matched each other (Supplementary Figure S2). Taken together, these results indicate that the bioinformatic analysis was correctly performed and that the wet experiments were also handled well. The mtDNA sequences obtained following the resequencing step were saved as FASTA files (Supplementary Data S1) and used to perform the subsequent analysis.

### 3.4. Phylogenetic Tree Analysis Confirmed the Similarity among Dogs

**Figure 5:**
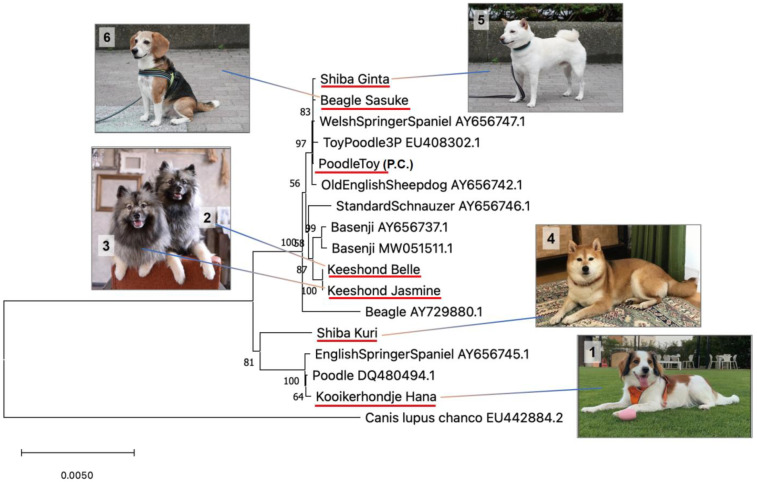
Phylogenetic analysis using whole mtDNA sequences. The numbers at the nodes indicate bootstrap support values.

Phylogenetic tree analysis was performed using the mtDNA sequences we obtained in this study, and the sequences were downloaded from a public database. Using the results, a tree was formed according to the similarities present. Furthermore, dogs No. 2 and 3 (Belle and Jasmine; daughter and mother) were depicted in the same position (Figure 5). These results suggest that the analysis was correctly performed, following the bioinformatics analyses presented in the aforementioned studies.

## 4. Discussion

In this study, whole mtDNA samples were sequenced using oral mucosal DNA obtained from six healthy dogs from four breeds bred and raised in Japan. Of these breeds, Keeshond and Kooikerhondje originate from the Netherlands; however, there are a few breeders of these rare breeds in Japan. The sequence information we acquired has been published in this paper (Supplementary Data S1) and is accessible to anyone anywhere in the world. Therefore, these sequence data would be useful for the diagnosis of dog mitochondrial diseases, the identification of individuals, and the performance of phylogenetics analyses in the future. In particular, the acquisition of the sequence data of rare breeds in Japan will be of great significance for the future development of dog welfare studies.

Few reports exist concerning the whole mitochondrial DNA sequences of dogs by applying NGS. Two studies conducted by Kowal et al. [29,30] sequenced whole mitochondrial DNA in mammary tumors and the whole blood of dogs to examine the relationship between genetic mutations and the progress of carcinogenesis using NGS. In that report, amplicon sequencing using LR-PCR was performed to amplify the whole mitochondrial DNA with two primer pairs with amplicon sizes of 9.5 and 9.8 kbp. The specimens were tumor tissue and blood, which must be collected invasively. In addition, the DNA sample were likely to be intact. In general, LR-PCR tends to yield a lower amplification efficiency; hence, partially degraded DNA may not yield PCR products. Therefore, it is necessary to use intact DNA to achieve successful results. However, in this study, we obtained good amplicons for four primer pairs using partially degraded oral mucosal DNA as a template. Each amplicon size was set as 4.7–5.7 kbp for the LR-PCR, an amount half of the amplicon size used in the above-mentioned study. Although the use of four primer pairs increased the complexity value, good PCR efficiencies were obtained despite the use of partially degraded DNA, and uniform coverage was obtained with no evidence of sample-to-sample variations. Therefore, this method is superior because it can be adapted to make use of partially degraded DNA using oral mucosal DNA, which can be collected non-invasively and could be generalized for use in dog medicine, phylogenetics, and forensic cases in the future.

The sequencing method established in this study for whole dog mtDNA might be applicable in forensic cases. Recently, in their study, Hsiou et al. reported that the resequencing performed for dog mtDNA obtained from a bite wound proved valuable in identifying perpetrators in cat-killing cases [31]; however, they also admitted that the method requires improvement, since the target region was limited and only 100 bp in dog mitochondrial cytochrome b was examined [32]. In this respect, the method has the advantage of being able to resequence whole mtDNA, which is expected to improve the sensitivity and specificity factors of DNA testing. Moreover, in addition to the species estimation, the method theoretically allows for the individual identification of each dog in comparison to the appropriate subject using oral mucosa samples. Since it is well known in the literature that the analysis of dog mtDNA can be used to successfully perform identifications in forensic cases [33,34], it can also be argued that the method presented in our study has the potential for use in crime scene investigations.

A recently published (2022) review paper [19] addressing mitochondrial diseases in dogs demonstrated several cases that could be useful for performing differential diagnoses in veterinary medicine. Cases of mitochondrial diseases in various dog breeds are discussed, and the diseases are divided into mitochondrial myopathies and encephalopathies. In addition, cases in which mtDNA mutations are also associated with tumors are presented. The examination of these cases was limited to the histological/biochemical examination of mitochondrial morphology/activity, and Sanger sequencing was deployed to examine targeted regions of mtDNA. Alone, these tests are considered insufficient for diagnosing mtDNA-related diseases and understanding their pathogenesis; there is a risk of misdiagnosis and incorrect treatment. For that reason, the authors [19] concluded that it is necessary to develop reliable molecular testing to unambiguously confirm the cause of the development of canine mitochondrial diseases [19]. The simple method for sequencing the whole mtDNA developed in this study using NGS may solve the above-mentioned problems. In future examinations aiming to determine mitochondrial disease in dogs, if the whole mtDNA can be read in addition to histological and biochemical tests, it may lead to a more accurate understanding of the disease and the development of new treatment methods.

This method is also expected to be applied in order to safeguard dogs; because mtDNA is maternally inherited, matching the mtDNA of mother and son/daughter dogs may lead to the identification of lost dogs. There is no doubt that there are many lost dogs in the world, but in Japan, if an owner cannot be found, the dog(s) may be put down. According to the latest statistical data from Japan’s Ministry of the Environment, between April 2022 and March 2023, there were 16,572 adult dogs and 4666 young dogs (totaling 21,238 dogs) who seemingly had no owners [35]. It is estimated that a considerable number of lost dogs were mixed in here, and since, 8402 dogs have been returned to their owners. In Japan, from June 2022, embedding a microchip into one’s dog to enable their individual identification became mandatory after the laws surrounding dog ownership were revised [36]. However, since this is still a new system, there are a significant number of dogs without microchips. Using the method established in this study, the whole mtDNA sequences of dogs No. 2 and 3 were found to be 100% identical, likely because they are mother and daughter (dogs No. 2 and 3, Supplementary Figure S2). Furthermore, the phylogenetic tree analysis also confirmed that the positions of the two dogs were completely the same. Therefore, the method can be used for maternal and child testing for dogs, and through the widespread incorporation of suitable maternal DNA in the field of animal welfare, owners may have more chance of finding their dog if it becomes lost. Furthermore, mtDNA is abundant in a single cell, making it easy to sequence. Even if a puppy is lost and its appearance has changed significantly after several years, if the owner has specimens such as hair, feces, or oral mucosa, it may be possible to identify the individual using our method. Similar methods, such as targeting mtDNA, have also been widely used in the field of forensic examination in humans, which enables the individual identification of missing persons in criminal investigations. Taken together, the method established in this study may enable the individual identification of dogs and positively contribute to their safeguarding.

Further attention should also be paid to the amount of data needed and the required size of the library insert. This is because the D-loop region in dog mtDNA contains short tandem repeats, which increases the risk of reduced mapping rates of reads obtained by NGS. Referring to the standard mtDNA sequence of dogs [21], the “TACACGTACG” sequence is repeated 30 times for a total of 300 bp in the D-loop region (base number: 15,458–16,727). Therefore, the insert size of 300 bp or higher for the library is considered desirable in the literature. However, the average insert size we achieved in this study was 170.47 bp (Supplementary Figure S3). Therefore, we were concerned that the coverage of short tandem repeats would be decreased in the D-loop region. Although accurate sequences were obtained in our results, decreased coverage values were confirmed in all samples. Therefore, the library preparation method needs to be improved slightly. The processes that need be improved, however, are the DNA fragmentation time and library size. If the average insert size can be optimized to 300 bp, it could improve the coverage of the D-loop region. Furthermore, the improved coverage can lead to cost reductions because the number of samples per NGS run can be increased.

In addition, setting a fixed target coverage value is also important in order to perform a successful experiment. In the aforementioned report presented by Kowal et al. [29], long amplicons (9.5–9.8 kbp) were obtained by LR-PCR, and the dog whole mtDNA was sequenced by NGS. However, a gap region was present; therefore, additional Sanger sequencing was performed to fill in the gap. Therefore, the use of NGS analysis alone was insufficient. Additionally, in the same reports, the coverage’s averaged value of 365–580× appears to be sufficient; however, there is a risk of inaccurate sequencing due to a possible loss of coverage in the D-loop region. In addition, repeated high-throughput experiments are always subject to the risk of various unpredictable experimental biases that depend on library preparation, pooling, NGS conditions, etc.; thus, coverage can vary widely from time to time. Therefore, it is important to set sufficient target coverage values at the outset to ensure consistently successful experiments. For our results, we were able to obtain accurate consensus sequences for all of the dogs and accurately determine the SNVs, even in the D-loop region (where coverage was reduced). Based on our results, it is reasonable to assume that a target coverage of at least 3000 would lead to a successful experiment, even if various biases were introduced in this method. However, increasing the target coverage also increases the sequencing cost, which requires good judgment and skills on the part of the scientist conducting the experiment. It is estimated that, if the researcher used the same reagents and NGS machine as in this experiment and set the acquisition coverage target to 3000, approximately 30–40 samples could be analyzed at once.

The DNA extraction process used in our experiment incorporated originally prepared reagents, which are inexpensive but complicated to use and time-consuming. This can be replaced by a commercial DNA extraction kit, which is simple, easy to use, and considerably reduces the labor required. Furthermore, in recent years, we have entered the era of laboratory automation, and robots are increasingly leading experimental operations. Nucleic acid manipulations, including PCR, are prone to human contamination, and there is a risk of obtaining non-specific PCR products. On the other hand, robots present almost no chance of contamination if the inside of the equipment is kept in a clean room. At present, we are verifying the method developed in this research using a robot that can automatically perform everything, from DNA extraction to library creation. The robot we are aiming to adapt is the LabDroid “Maholo” [37]. It was created by a Japanese company (Robotic Biology Institute Inc., Koto-ku, Tokyo, Japan) and utilizes car manufacturing technology. Because the Maholo is good at performing high-throughput experiments with nucleic acids, considering its simplicity, the method proposed in this study could be easily implemented by the Maholo. If this method is combined with automation via the Maholo, it could be very useful for further research on dog medicine and welfare.

The four primers used in this study were highly specific, presenting no non-specific amplifications for all of the dog DNA samples. This result suggests that the primer pairs are universal primers that can be applied to other dog breeds. As for the specimen types, it can be argued that they can be applied to intact DNA derived from the blood and tissue of dogs. However, caution should be exercised when using DNA that is considerably degraded, e.g., DNA obtained from FFPE, necrotic tissue, hair, feces, urine, and infected tissue. Since this primer performs LR-PCR, whether it can be applied to highly degraded DNA is not certain. Moreover, if a large amount of contaminated DNA is obtained from other species (contaminated due to, for example, feces or infected tissue), this would be a factor that would reduce PCR specificity. Therefore, further investigations are required to determine if this method can be adapted to samples other than oral mucosal DNA.

There were some evident limitations when applying this method to test for mitochondrial diseases in dogs. Deletion-type mutations in human mitochondrial diseases can cause rare, large deletions [11]. If a sequence of primer pairs exist in the deletion region, PCR amplification cannot be performed; hence, there is a possibility that large deletions cannot be detected. Therefore, it is necessary to verify the PCR amplicons and, if unobtainable, consider the application of different strategies. In addition, if SNVs are present in the sequences bound by these primers, it may lead to a decrease in PCR efficiency and insufficient amplicons. Therefore, researchers should always be prepared to use alternative methods, such as using other primer pairs or switching to the capture method.

## 5. Conclusions

By using oral mucosal DNA adjusted to 1 ng/μL as a template with four primer pairs and NGS, we were able to accurately sequence whole mtDNA obtained from six dogs from four different breeds. Based on the results of this study, we believe that the proposed method has the potential to provide new insights into this study area and facilitate advancements in the research being conducted on dog medicine and welfare.

## Figures and Tables

**Table 1:** Summary of the variants.

Dog No.	Dog Breed/Gender	Total Length (bp) of mtDNA	Number of Variants			Coverage of Variants	Number of Non-Synonymous Amino Acids	Pathogenic Variant
1	Kooikerhondje/female	16,730	SNV	101	Total: 104	6135 to 51,966 (Average: 20,178)	17	N/A
			MNV	1				
			Insertion	2				
			Deletion	0				
2	Keeshond/ female	16,732	SNV	33	Total: 36	6525 to 24,492 (Average: 13,442)	4	N/A
			MNV	0				
			Insertion	3				
			Deletion	0				
3	Keeshond/ female	16,732	SNV	33	Total: 36	7978 to 33,320 (Average: 17,579)	4	N/A
			MNV	0				
			Insertion	3				
			Deletion	0				
4	Shiba Inu/ female	16,730	SNV	105	Total: 109	6300 to 45,580 (Average: 17,999)	20	N/A
			MNV	0				
			Insertion	3				
			Deletion	1				
5	Shiba Inu/ male	16,730	SNV	23	Total: 25	12,637 to 36,862 (Average: 21,158)	3	N/A
			MNV	0				
			Insertion	2				
			Deletion	0				
6	Beagle/ male	16,730	SNV	21	Total: 23	9690 to 44,299 (Average: 22,209)	3	N/A
			MNV	0				
			Insertion	2				
			Deletion	0				
P.C.	Toy Poodle/ male	16,730	SNV	27	Total: 29	9122 to 28,966 (Average: 17,035)	3	N/A
			MNV	0				
			Insertion	2				
			Deletion	0				

P.C.: positive control, SNV: single-nucleotide variant, MNV: multiple-nucleotide variant, N/A: not applicable.

## Data Availability

The FASTQ files obtained from NGS were deposited in the “Sequence Read Archive (SRA; https://www.ncbi.nlm.nih.gov/sra/; accessed on 13 March 2023)” under accession number: PRJNA943940. The whole mtDNA sequences of the dogs featured in this study are available in the Supplementary Materials (Supplementary Data S1).

## Supplementary Materials

The following supporting information can be downloaded at: https://www.mdpi.com/article/10.3390/ani13142332/s1, Figure S1: Quality scores for resequencing analysis in CLC software; Figure S2: Alignment analysis of two dogs that are parent and child; Figure S3: Insert sizes calculated from the mapped reads; Table S1: The primer sequences for whole mtDNA of dogs; Table S2: Variant detection for dogs; Data S1: FASTA files of whole mtDNA sequences of dogs.
