# Supplementary material for: Establishing a Sequencing Method for the Whole Mitochondrial DNA of Domestic Dogs
Source: Animals (Basel). 2023 Jul 17;13(14):2332. doi: 10.3390/ani13142332 (PMC10375980; doi:10.3390/ani13142332)
Supplement: Supplementary file 1 [file animals-13-02332-s001.zip › Supplementary_files_sugasawa_et_al/Figure_S1.pdf]

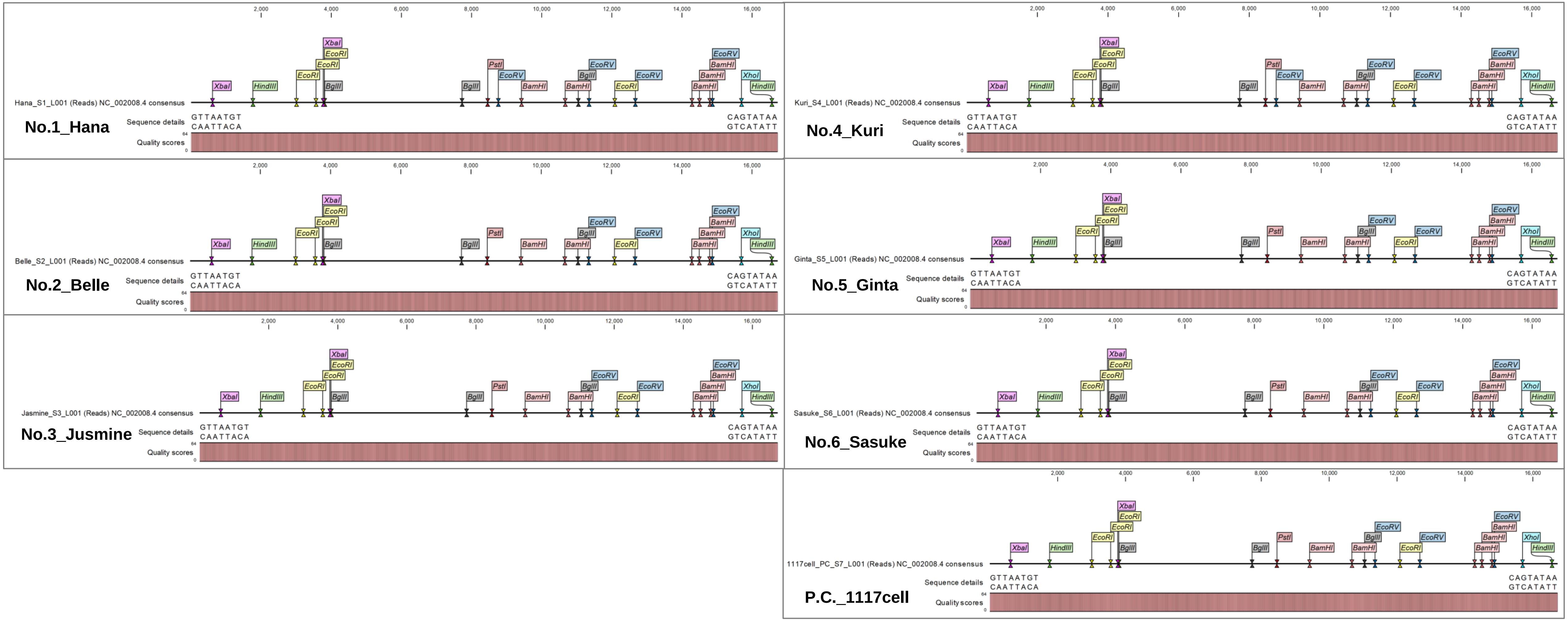

Figure S1. Quality scores for resequencing analysis in the CLC software.
The bar plots of pink color show the quality score for each base position, with the highest value of 64 for all base positions.
