# Supplementary material for: Establishing a Sequencing Method for the Whole Mitochondrial DNA of Domestic Dogs
Source: Animals (Basel). 2023 Jul 17;13(14):2332. doi: 10.3390/ani13142332 (PMC10375980; doi:10.3390/ani13142332)
Supplement: Supplementary file 1 [file animals-13-02332-s001.zip › Supplementary_files_sugasawa_et_al/Figure_S2.pdf]

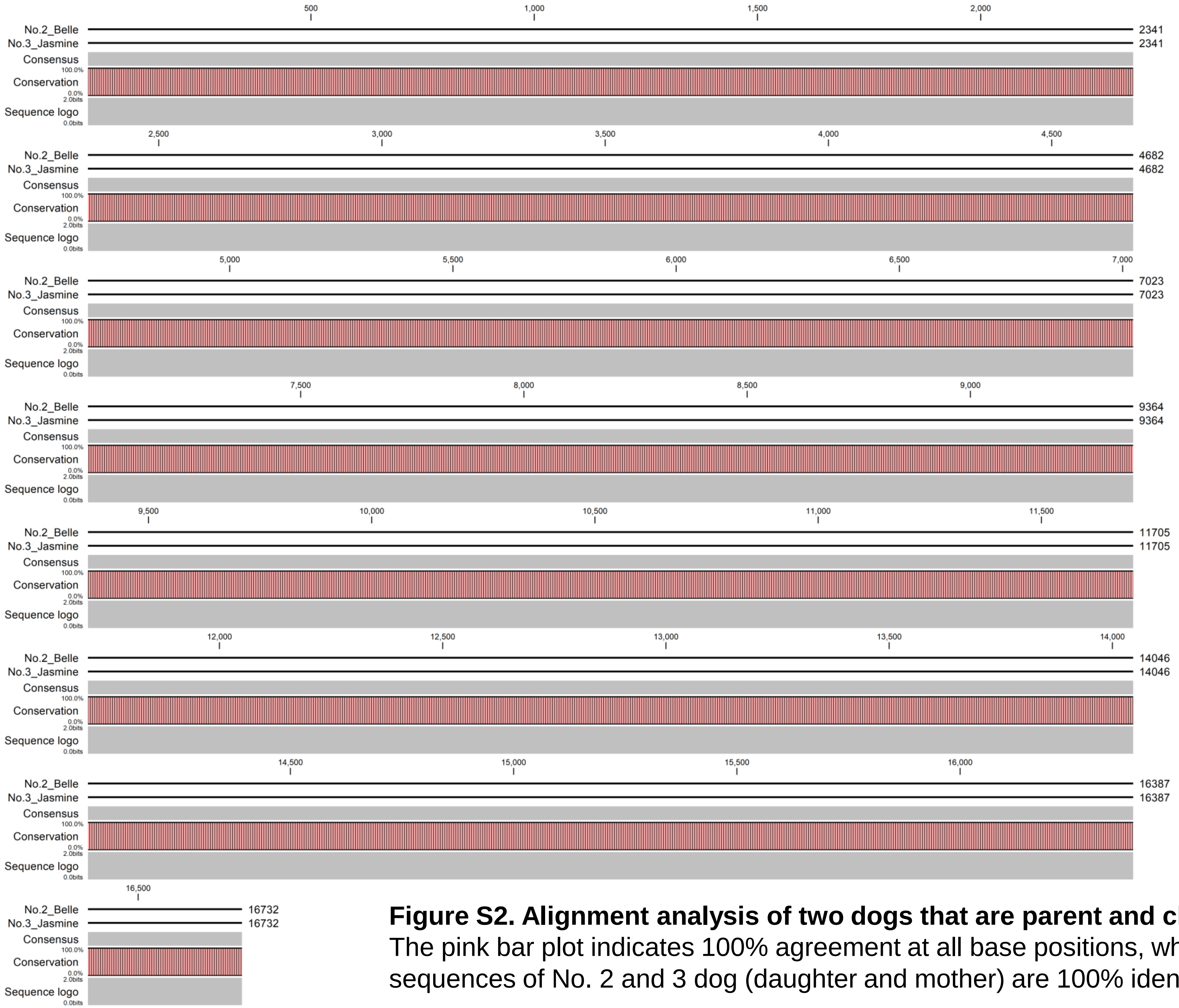

Figure S2. Alignment analysis of two dogs that are parent and child.
The pink bar plot indicates 100% agreement at all base positions, which mean that whole mtDNA sequences of No. 2 and 3 dog (daughter and mother) are 100% identical.
