# Supplementary material for: Establishing a Sequencing Method for the Whole Mitochondrial DNA of Domestic Dogs
Source: Animals (Basel). 2023 Jul 17;13(14):2332. doi: 10.3390/ani13142332 (PMC10375980; doi:10.3390/ani13142332)
Supplement: Supplementary file 1 [file animals-13-02332-s001.zip › Supplementary_files_sugasawa_et_al/Figure_S3.pdf]

1.5 Mapped paired reads

The table is based on 7 samples.

Sample name	Paired reads (%)	Broken reads (%)	Mean distance	Standard deviation
Sasuke_S6_L001 (Mapping report)	98.84	1.16	171.69	76.21
1117cell_PC_S7_L001 (Mapping report)	98.84	1.16	171.01	76.26
Belle_S2_L001 (Mapping report)	98.83	1.17	167.33	73.83
Kuri_S4_L001 (Mapping report)	98.62	1.38	171.88	76.66
Jasmine_S3_L001 (Mapping report)	98.64	1.36	170.62	77.07
Hana_S1_L001 (Mapping report)	98.92	1.08	170.43	75.86
Ginta_S5_L001 (Mapping report)	98.73	1.27	170.29	75.08
Minimum	98.62	1.08	167.33	73.83
Median	98.83	1.17	170.62	76.21
Maximum	98.92	1.38	171.88	77.07
Mean	98.77	1.23	170.47	75.85
Standard deviation	0.11	0.11	1.51	1.09

Figure S3. Insert sizes calculated from mapped reads.
Red underline indicates average insert size from all samples.
